# Clinical characteristics and outcomes of surgery for renal artery aneurysms: analysis from a single institute in Japan

**DOI:** 10.1007/s00595-025-03146-3

**Published:** 2025-10-13

**Authors:** Yu Tadokoro, Katsuyuki Hoshina, Kazuhiro Miyahara, Masaru Kimura, Takuro Shirasu, Toshio Takayama

**Affiliations:** Division of Vascular Surgery, Department of Surgery, Graduate School of Medicine, The University of Tokyo, 7-3-1, Hongo, Bunkyo-Ku, Tokyo 113-8655 Japan

**Keywords:** Renal artery aneurysm, Surgical outcomes, Expansion rate

## Abstract

**Purpose:**

We investigated the clinical characteristics of renal artery aneurysm (RAA) and the outcome of surgical intervention.

**Methods:**

The subjects of this retrospective analysis were 105 patients who were admitted to our department between 1999 and 2023.These 105 patients had a collective total of 151 RAAs.

**Results:**

The RAAs were localized unilaterally in 89 patients and bilaterally in 16 patients. The average age at diagnosis was 61.8 ± 12.4 years. The mean diameter on admission was 14.9 ± 7.0 mm. The expansion rate was 0.055 ± 0.46 mm/month (mean follow-up period: 53.1 months). “Egg-shell” calcification appearance was found for 21% of the RAAs, with a lower expansion rate (0.0005 ± 0.003 mm/month). Rupture occurred in one patient with a pseudoaneurysm. 20 aneurysms co-existed with other aneurysms, most commonly with splenic aneurysms (*n* = 10). Surgery was performed for a collective 34 RAAs in 24 patients, including 15 aneurysmectomies with or without bypasses, 8 aneurysmorrhaphies, 2 ex vivo aneurysm repairs, 1 coil embolization, and 1 stent graft insertion. Post-operative complications included renal infarction (*n* = 8) and bypass occlusion (*n* = 4), with the estimated glomerular filtration rate decreasing to 80% (range: 67–99%) and 76% (55–77%), respectively.

**Conclusion:**

RAAs have low expansion rates and a minimal risk of rupture, particularly if they are of the “egg-shell” type. Post-operative adverse events included renal infarctions and bypass failure, with mild renal impairment.

## Introduction

Renal artery aneurysms (RAAs) are the second most common visceral type of aneurysm, after splenic artery aneurysms [1]. They are relatively rare, with an overall incidence of 0.01–0.1% in the general population [1–5] and thiers natural history is not completely understood. Vascular surgeons have debated the threshold of the diameter of an aneurysm that warrants surgical or endovascular intervention. The guidelines published by the Society for Vascular Surgery (SVS) in 2020 resolved this issue to an extent, suggesting treatment particularly for aneurysms larger than 3 cm in patients with uncomplicated RAAs and acceptable operative risk [1]. However, given that the recommendation grade is 2C, the indication should be decided on a case-by-case basis. Based on our experience, we hypothesized that risk of expansion and rupture of RAAs might be lower depending on their specific characteristics. We conducted this study to investigate the natural history and characteristics of RAAs, and to assess the outcomes of surgical intervention in a single Japanese institute.

## Methods

### Patient cohort

RAAs are defined as focal, isolated dilatations of all three layers of the arterial wall, measuring more than 1.5 times the diameter of the adjacent disease-free proximal arterial segment. In this study, RAAs were identified as focal dilatations greater than 5 mm of the renal artery. This definition is based on the minimum diameter of RAAs diagnosed through imaging findings in our department, where they are either monitored or treated. This diameter was consistently 5 mm, aligning with the minimum diameter of RAAs reported in previous studies. [6] Using the National Clinical Database disease code, including “aneurysm of the renal artery,” we identified 125 patients with RAA at this single institution during the 25 years from 1999 to 2023. Patients registered in the database in duplicate, those not diagnosed with an RAA, and those who did not receive consultations or were not diagnosed in our department were excluded from the analysis. Demographic data, comorbidities, and other clinical data were collected retrospectively from medical records.

### Imaging data and growth rate calculation

**Fig. 1:**
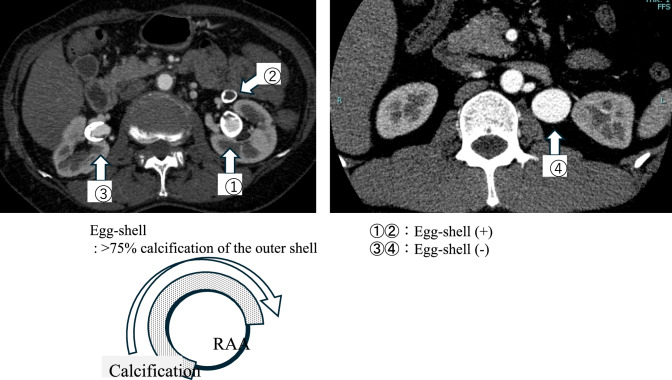
Definition of egg-shell calcifications and representative cases with and without egg-shell calcifications

Cross-sectional imaging studies were reviewed to collect anatomic data, including aneurysm laterality, the presence of other aneurysms, RAA location (right, left, or bilateral), aneurysm morphology (saccular or fusiform), aneurysm calcification (“egg-shell” calcification was defined as > 75% calcification of the outer shell), and maximum aneurysm diameter (Fig. 1). The aneurysm diameter was measured directly from one outer wall to the opposite outer wall, as the maximum short diameter for fusiform aneurysms and the long diameter for saccular aneurysms. The expansion rate was calculated using computed tomography (CT) images at more than two time points as follows:

$$\frac{{{\mathrm{recent}} {\mathrm{diameter}} - {\mathrm{initial}} {\mathrm{diameter}}}}{{{\mathrm{follow}} - {\mathrm{up}} {\mathrm{period}}}}$$

The Ethics Committee of the University of Tokyo Hospital (no. 3316-(6), 3252-(5)) approved the imaging data and electronic clinical data being used for this study.

### Statistical analysis

JMP Pro 13 software (SAS Institute Inc. Cary, NC, USA) was used for all statistical analyses. Categorical variables are presented as numbers and percentages, and continuous variables are presented as medians and standard deviations. Differences between groups were analyzed using Student’s *t* test. Multiple linear regression models were calculated in the sensitive analysis for modified expansion rate. For each of the analyses, a *P* value of < 0.05 was defined as significant.

## Results

### Patients

Between 1999 and 2023, 125 patients with RAAs were identified in our hospital database (tracking rate, 84%). The subjects of this analysis were 105 of these patients with a collective total of 151 RAAs, who met the study inclusion criteria. The average age on admission was 61.8 ± 12.4 years (range, 27–89), and 54% of the patients were women. Concomitant diseases included hypertension (56%), diabetes mellitus (12%), and dyslipidemia (27%). Notably, there was a high incidence of current or former smokers (42%) (Table 1). Co-existing aneurysms were most commonly identified at the splenic artery (*n* = 10), followed by the common iliac (*n* = 4), hepatic (*n* = 4), celiac (*n* = 3) arteries, internal iliac artery (*n* = 2), and cerebral arteries (*n* = 2), as well as at other arteries including the carotid and lateral thoracic arteries, ascending and abdominal aortae, and superficial femoral artery (*n* = 1, each). Multiple aneurysms were found in seven patients (Table 1). Co-existing vascular lesions were likewise identified with renal artery stenosis (*n* = 5), renal arteriovenous fistula (*n* = 4), celiac artery stenosis (*n* = 2), superior mesenteric artery stenosis (*n* = 2), pelvic arteriovenous fistula (*n* = 2), and others (*n* = 1), including celiac artery dissection, subclavian artery stenosis, and thoracic aortic dissection (Table 1).

**Table 1:** Clinical characteristics of the patients

	Average ± SD (cases)
Age (years)	61.8 ± 12.4 (*n* = 105)
Women	57/105 (54%)
< Concomitant diseases >	
Hypertension	54/95 (56%)
Diabetes mellitus	12/94 (12%)
Dyslipidemia	29/92 (27%)
Coronary artery disease	4/92 (4%)
Cerebrovascular disease	4/92 (4%)
Collagen disease	3/92 (3%)
History of trauma	2/92 (2%)
Current/ex-smoker	35/82 (42%)
Family history of aneurysms or arterial dissection	9/70 (12%)

Characteristics of the renal artery aneurysms	
< *Location* >	
Right	56/105 (53%)
Left	33/105 (31%)
Bilateral	16/105 (15%)
< *Shape* >	
Saccular	136/151 (90%)
Fusiform	12/151 (8%)
Pseudoaneurysm	1/151 (1%)
Unknown	2/151 (1%)
< *Morphology* >	
Egg-shell calcification	33/151 (21%)
< *Diameter, periods* >	Median or mean ± SD (range)
At admission	14.9 ± 7.0 mm (5–40 mm)
The most recent timepoint	15.2 ± 6.7 mm (5–40 mm)
Follow-up period (*n* = 142)	35 ± 57.3 months (0–201 months)
Expansion rate (*n* = 120)	0.055 ± 0.46 mm/month (0–5 mm/month)
of egg-shell (*n* = 33)	0.0005 ± 0.003 mm/month (0–0.02 mm/month)
< *Co-existing aneurysms* >	Number of cases
Splenic artery	10
Hepatic artery	4
Common iliac artery	4
Celiac artery	3
Internal iliac artery	2
Cerebral artery	2
Others*	1
Multiple co-existed	7
< *Co-existing vascular lesions* >	Number of cases
Renal artery stenosis	
Ipsilateral	2
Bilateral	3
Renal arteriovenous fistula	4
Celiac artery stenosis	2
Superior mesenteric artery stenosis	2
Pelvic arteriovenous fistula	2
Others**	1

*Included the carotid artery, lateral thoracic artery, ascending aorta, abdominal aorta, and superficial femoral artery

**Included celiac artery dissection, subclavian artery stenosis, and thoracic aortic dissection

### Aneurysm characteristics

**Fig. 2:**
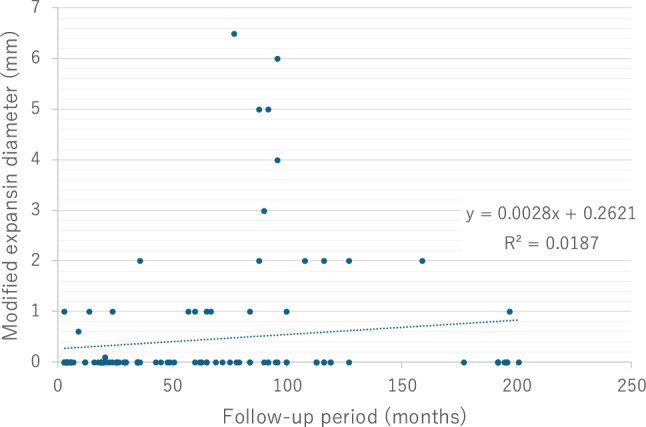
Correlation between the expansion diameter and follow-up periods

Half of the RAAs were located on the right side (53%) ipsilaterally, and 15% were located bilaterally. Multiple RAAs were present in 26% of the patients. The mean diameter of the RAAs was 14.9 ± 7.0 mm (range: 5–40 mm) on admission, which increased to 15.2 ± 6.7 mm at the most recent follow-up. The median observation period was 35 ± 57.3 months (range: 0–201 months) and the average expansion rate was 0.055 ± 0.46 mm/month. Most RAAs had no evidence of substantial growth during the observation period. We considered RAAs showing expansion during a follow-up period of less than 3 months to be inappropriate for observing the natural history of RAA expansion, and we performed a sensitivity analysis after excluding them as outliers. In this analysis, the correlation of expansion rates in RAA cases was expressed as the equation: y = 0.0028x + 0.2621 (R^2^ = 0.0187) (Fig. 2). Most RAAs had a saccular morphology (90%) and 33 (21%) of aneurysms had an “egg-shell” calcification appearance. Although no significant differences were observed, the expansion rate was even lower in patients with “egg-shell” calcification (0.0005 ± 0.003 mm/month, *p* value = 0.098) (Table 1). Most of the RAAs exhibited no substantial growth during the observation period. The patients remained asymptomatic during follow-up. Rupture occurred in only one patient with a renal artery pseudoaneurysm coexisting with a superficial femoral artery pseudoaneurysm, but they did not suffer shock. No surgical intervention was performed in any patient with “egg-shell” calcification.

### Surgical intervention for RAAs

Surgical interventions were performed in 24 patients with a collective total of 34 RAAs. The mean largest aneurysm diameter of the RAAs in patients with multiple RAAs was 24.6 ± 6.8 mm (range: 15–40 mm), measured during surgery. There was one case of symptomatic pseudoaneurysmal rupture with symptoms. The mean age, proportion of women, and percentage of concomitant diseases were similar to those in the overall population although a lower rate of current or former smokers (42% vs. 27%) was observed (Table 2).

**Table 2:** Characteristics of the patients with renal arterial aneurysms (RAAs) treated surgically (34 RAAs in 24 patients)

(a)	
Age (years)	58.3 ± 10.9 (*n* = 24)
Women	13 (54%) (*n* = 24)
< *Concomitant diseases* >	
Hypertension	11/23 (47%)
Diabetes mellitus	2/23 (8%)
Dyslipidemia	5/23 (21%)
Coronary artery disease	1/23 (4%)
Cerebrovascular disease	1/23 (4%)
Collagen disease	1/23 (4%)
History of trauma	0/23 (0%)
Current smoking	6/22 (27%)
Family history of any aneurysms or arterial dissection	1/23 (4%)

	Mean ± SD (range)
*Characteristics of Surgically intervened RAAs (34 RAAs in 24 cases*)	
Maximum aneurysm diameter	24.6 ± 6.8 mm (15–40 mm)
< Operative details >	
Aneurysmectomy with renal artery reconstruction (bypass)	*n* = 15
Aneurysmorrhaphy	*n* = 8
Ex vivo aneurysm repair	*n* = 2
Coil embolization	*n* = 1
Stent graft	*n* = 1
[Simultaneous multiple repair]	*n* = 3

Aneurysmectomy with renal artery reconstruction, including bypass, was performed in 15 patients, and aneurysmorrhaphy was performed in 8 patients. Ex vivo aneurysm repair was performed in two patients, whereas endovascular repair using a stent graft or coil embolization was performed in two others (Table 2).

### Post-operative adverse events

Post-operative complications developed in 16 (67%) patients as renal infarction in eight patients, renal artery stenosis in five, and bypass occlusion in four. The range of infarction was classified using computed tomography (CT) images in 25% increments of the total kidney volume. The infarcted lesions were within 25% in five of the eight patients (Table 3).

**Table 3:** Post-operative adverse events and changes in eGFR

	Cases*	Change of eGFR (range)
Renal infarction	*n* = 8	80% (67–99%)
Infarct volume of the kidney		
< 25%	*n* = 5	
25 ~ 50%	*n* = 1	
51 ~ 75%	*n* = 1	
76% <	*n* = 1	
Renal artery (anastomotic) stenosis	*n* = 5	No change
Post-operative hypertension	*n* = 4	No change
Bypass graft occlusion	*n* = 4	76% (55–77%)
Bypass graft dilatation	*n* = 2	No change
Ileus	*n* = 1	No change

*Cases were duplicated

### Changes in estimated glomerular filtration rate (eGFR)

To reveal the effect of infarction or graft occlusion on renal function, we analyzed changes in eGFR. The eGFR decreased to 80% (range: 67–99%) of the preoperative level in the eight patients with infarction and to 76% (range: 55–77%) in the four patients with bypass graft occlusion cases (Table 3).

### A case of complex bypass reconstruction

**Fig. 3:**
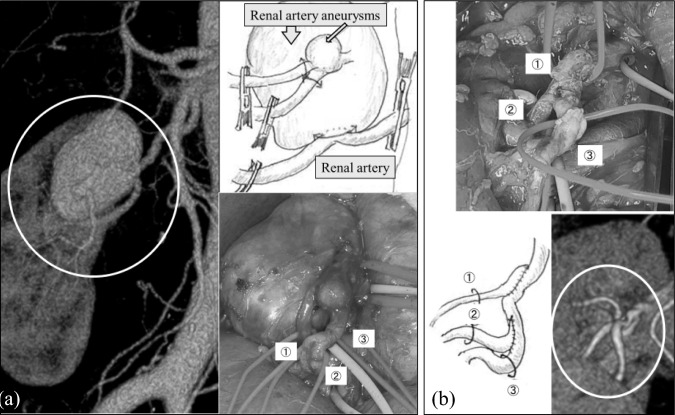
A case of multiple renal arterial aneurysms treated with surgical intervention

We summarize the case of a complex bypass (Fig. 3). The patient was a 63-year-old woman with no deterioration of renal function. CT revealed two RAAs (32 mm and 6 mm in diameter) with one afferent renal artery and three efferent branches. Following RAA resection, the small branches were anastomosed to the trunk of the main renal artery. The clamp time was 39 min. Based on our experience with bypass graft occlusions, we confirmed the anastomotic morphology carefully, ensuring that the graft was not twisted or kinked, particularly after removing the towel beneath the kidney that had been inserted to aid surgical exposure (Fig. 3). The bypass has remained patent for 5 years.

## Discussion

The timing of surgical intervention for RAAs has not been established because of our limited understanding of the natural history of RAA, including the risk of rupture and outcomes after intervention. The SVS guideline published in 2020 provided an unconvincing recommendation for intervention for uncomplicated RAAs larger than 3 cm in diameter in patients with acceptable operative risk [1]. Before the publication of this guideline, the commonly accepted indication for intervention was an RAA > 1.5–2 cm in diameter [7–9]; however, recent reports showed evidence that a 2.0 cm threshold for intervention was too aggressive [6, 10]. Klausner et al. reported that there was a low risk of rupture of RAAs even when the diameter was > 2 cm and noncalcified, that RAA growth rate is less than 0.09 cm/y, and that calcification does not protect against growth [10]. As previous large studies on RAAs showed no rupture [10, 11], RAAs appear to have a low risk of rupture, except in patients who may give birth.

This study revealed a low expansion rate, similar to that of previous reports (0.6–0.8 mm/year) [6, 10]. Moreover, by focusing specifically on RAAs with “egg-shell” calcification, as defined by the report on splenic artery aneurysms, we observed that the rupture risk was even lower. Sano et al. reported that the egg-shell appearance of splenic artery aneurysms was an independent inverse risk factor affecting the splenic artery aneurysm dilatation rate. The claim that severely calcified RAAs have a low risk of rupture remains controversial [10]. We referred to the “egg-shell” definition from a report analyzing splenic artery aneurysms [12]. However, we could not identify significant differences between RAAs with and those without “egg-shell” calcification because of our small sample size. Egg-shell calcification has been referred to only in relation to these visceral artery aneurysms. Unlike other aneurysms, these two aneurysms have the diameter threshold of surgical intervention as per the guidelines [1], indicating a potentially lower risk of rupture.

The characteristics of RAAs in our cohort were similar to those of other single-arm retrospective studies, with an average age of approximately 60 years, a high rate of hypertension, and a relatively high proportion of women [13]. The unique features of RAAs in our study were high rates of current and former smokers and a clearly defined “egg-shell” calcification appearance. The low expansion and rupture risks of RAAs with “egg-shell” calcifications identified in this study could support the clinical decisions regarding intervention.

Several postoperative complications might occur during long-term follow-up. Renal impairment (as indicated by a decrease in eGFR) was mild, even after bypass graft occlusion or massive infarction. However, as these might be procedure-related adverse events, we reviewed the intraoperative findings and CT images regularly to determine the cause. We speculate that graft kinking after anastomosis might be associated with occlusion. As shown in the case of the complex reconstruction summarized in this manuscript, a detailed operative plan is necessary, ensuring no issues with the morphology of the reconstruction and carefully checking for kinks and stenosis before abdominal closure.

The present study has some limitations. Its retrospective and single-center design might cause selection bias. The small number of patients could not provide definitive statistical evidence, particularly for rupture rates as there was only one case of rupture. This might cause bias when calculating the expansion rates for patients with various follow-up periods. Additionally, we did not analyze women of childbearing age separately because the sample size made this impossible. Furthermore, we analyzed the patients with long-term follow-up. In recent decades, more advance endovascular devices and techniques have been developed, and our surgeons are skilled in using them, while following the endovascular-first strategies according to the guidelines. Considering that the open-first strategy has been recently reevaluated, we now opt for open surgery, depending on the location of the RAAs. [14, 15] More than 90% of the surgical procedures in this study were performed before the guidelines were revised, which might have led to a bias in choosing the surgical procedures, with a significantly lower rate of endovascular treatment selection. Moreover, the early cases in this study included RAAs that at the time were defined as those with an arterial diameter > 5 mm, which might have led to underestimation of the mean RAA diameter and expansion rate.

In conclusion, our findings suggest that RAAs have low expansion rates and a minimal risk of rupture. “Egg-shell” calcifications were present in 21% of our RAA cohort, with expansion rates even lower than those of RAAs without calcifications. Post-operative adverse events included renal infarctions and bypass failure, with mild renal impairment. Although the surgical indication for RAA has been based on its size and shape, we should also consider that the RAA tends to expand slowly and that the risk of rupture is low in the "egg-shell" types.
